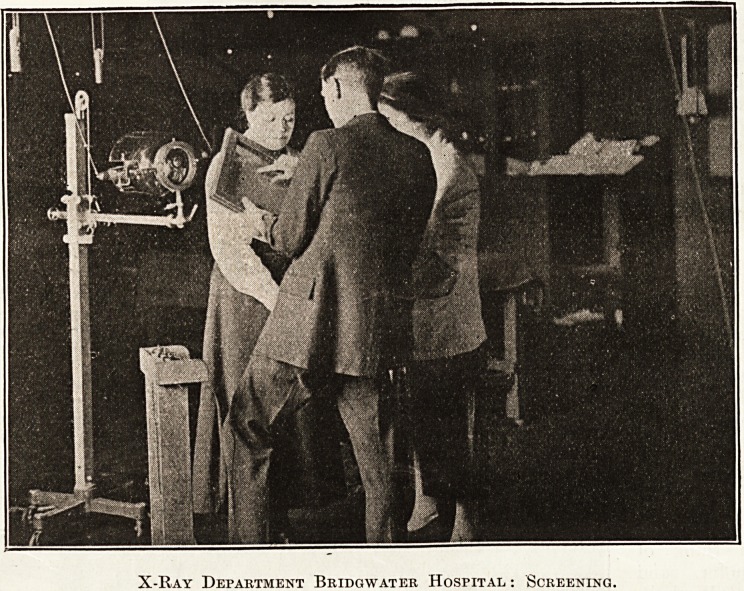# How a Country Hospital Was Saved

**Published:** 1923-08

**Authors:** Walter Deacon


### HOW A COUNTRY HOSPITAL WAS SAVED.

### "LET THE PEOPLE KNOW."

By WALTER DEACON.

AT a time when so many of our hospitals are at their wits' end for money and haunted by the fear of closed wards, it may be of interest to relate how a hospital in a country town has faced and overcome the difficulty. In March last the treasurer of the Bridgwater Hospital announced that the debt had reached £2,500, and the committee realised that a determined effort must be made to clear it, and so avoid otherwise inevitable disaster.

THE MEDICAL AND NURSING STAFFS OF BRIDGWATER HOSPITAL.

But how? It is no easy matter to raise a large sum of money to pay a debt anywhere at present, and in a district mainly agricultural the problem might well be deemed hopeless. Nevertheless, we have done it, and I will briefly describe our effort and triumph. We determined to "let the people know," and they came to our help gladly—yes, even eagerly. A campaign of enlightenment was planned out. The first step was to prepare a set of lantern slides illustrating the daily work of the Hospital. Some three dozen photographs of actual ward and theatre work were taken and a popular lecture written to explain the slides and show the actual work a hospital does.

The first lecture was given in the Town Hall of Bridgwater, with the Mayor in the chair, and proved a wonderful send off to a campaign carried into every village in the district served by the Hospital. No money was appealed for at the lectures, only collections to cover the cost of the lantern, etc., but we "let the people know," and the success of the money-raising effort which followed was largely attributed to the lantern campaign. It will be useful perhaps to say what form the lecture took. The slides illustrated a detailed tour of the building. The lecturer gave a brief history of the Hospital, and after pictures of the entrance hall and the matron's office had been shown, the men's ward was entered and details of the day's work were illustrated, such as a steam tent in use, the house surgeon putting a plaster of Paris bandage on a fractured leg, a nurse taking a child's temperature, etc. A slide showing a patient being conveyed in the electric lift brought us to the women's ward and pictures of the work were again shown—staff nurse giving oxygen, sister adjusting dressings, application of a woman's splint, etc.

Several slides were next introduced of children in hospital. Then the sterilizing room was illustrated, which gave a chance for a little talk on aseptic methods before slides dealing with the operating theatre were shown. No part of the lecture excited greater public interest than the slides illustrating an operation and details of theatre work. At this point statistics were fired off—numbers of the in-patients, out-patients, casualties, operation X-ray and electrical patients, showing the mass of work attacked during the year. After slides of the electrical and massage departments had been shown

The Theatre Ready for an Operation.

A new spirit pervaded the district, and languid approval of the Hospital was transformed into such enthusiasm that when the eventful week arrived an elaborate programme was carried out with all classes and sections working together without hitch or friction. On the Sunday, churches and chapels gave special collections; on Monday a big dance was held; on Tuesday there was a matinée at the local theatre; on Wednesday there was a Rose Day and daylight procession; and on Thursday, Friday and Saturday we held the big Country Fair which all had been working for. The result was a triumph. They asked for £2,000; they gave us over £3,200, and to-day the Hospital is free.

further details of daily routine followed. Slides from the X-ray department showing treatment, taking skiagrams and actual results always caused intense interest and lent themselves admirably to lantern propaganda. The village audiences saw for the first time the capacities of this new branch of work at the Hospital.

Finally the direct appeal was made, and the lecturer ceased speaking while a slide appeared on the screen exhibiting an open pass book revealing a £2,500 overdraft. Other slides said "Will you help?"; "While you enjoy health think of those who suffer," and "Let Hospital Week be your thanksgiving week." Throughout the lectures humorous and pathetic stories of actual life in the local Hospital brought home to the listeners that the Institution existed to help them and merited their help in return. The result of the lecture campaign was nothing less than dramatic. Town and village rose to the occasion, Hospital Week Committees were formed and offers of help poured in—one village promised a stall which has produced £337; other villages arranged house-to-house collections, one village collecting £65, another £45, and so on.

[The following is a key to the personages in our group of the medical and nursing staff. Names from left to right:—*Back Row.*—Nurse Hickman, Nurse Jones, Nurse Gage, Nurse Wiltshire, Nurse Callen, Nurse Thorne, Nurse Turner, Nurse Shewan. *Middle Row.*—Nurse Stallard, Miss Harrison (Dispenser), Nurse Arnott, Sister Emberson, Sister Dowding, Sister Huxtable, Mrs. Mogg (Masseuse), Nurse Coombs, Nurse Kearns, Nurse Thorp, Nurse French Smith. *Front Row.*—Dr. O'Dwyer Thomas (House Surgeon), Dr. Wilberforce Thompson, Dr. H. R. Routh, Miss Kitching (Matron), Dr. Harvey Bird, Dr. R. Coates, Mr. W. Deacon (Radiographer).]

Taking a Dental Skiagram.

X-Ray Department Bridgwater Hospital: Screening.

## Figures and Tables

**Figure f1:**
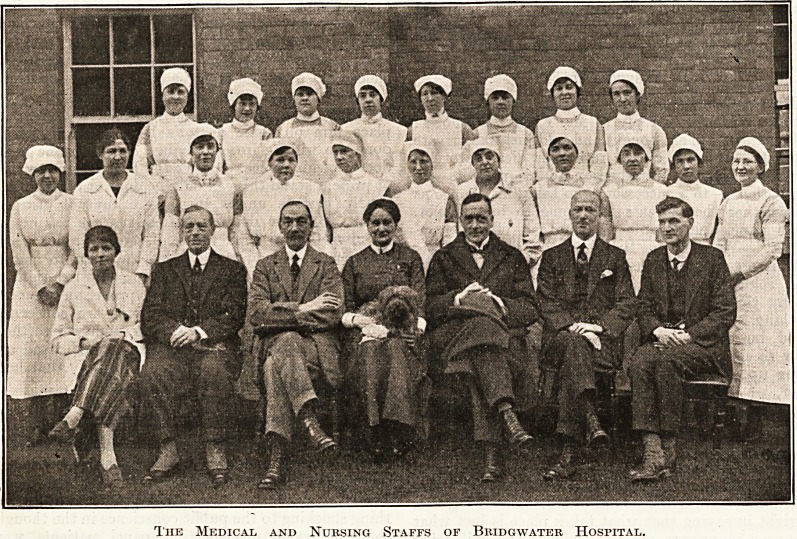


**Figure f2:**
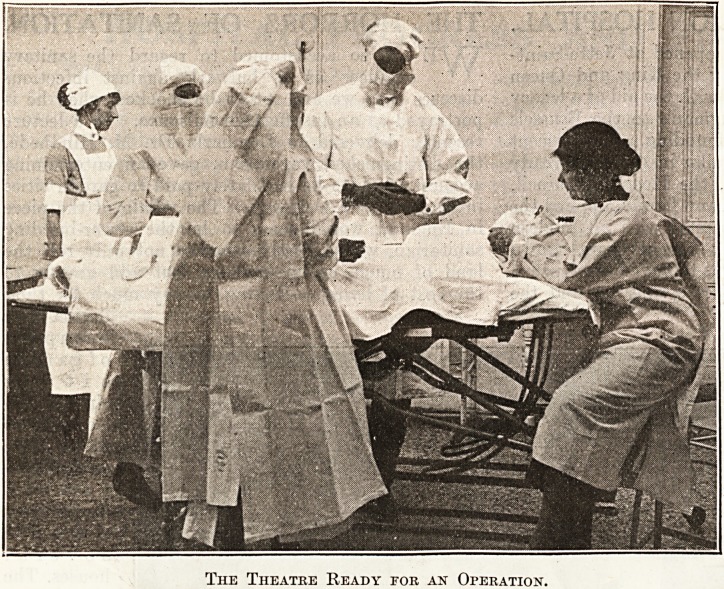


**Figure f3:**
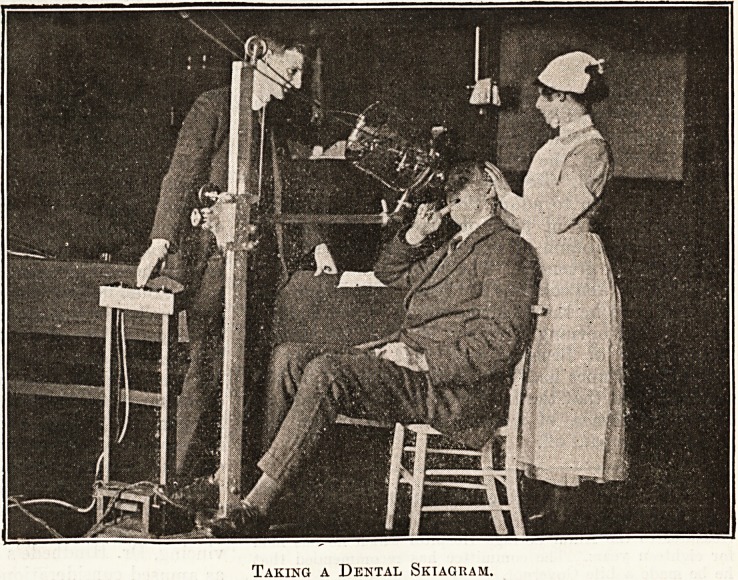


**Figure f4:**